# Development and validation of a nomogram for prediction postoperative sleep disorders in patients with oral cancer: a prospective study

**DOI:** 10.3389/fonc.2026.1739508

**Published:** 2026-04-21

**Authors:** Ruyue Qiu, Yunyu Zhou, Guangman Wang, Jingya Yu, Yajun Li, Liyan Mao, Grace Paka Lubamba, Xiaoqin Bi

**Affiliations:** 1 State Key Laboratory of Oral Diseases & National Center for Stomatology & National Clinical Research Center for Oral Diseases & Department of Orthognathic and Temporomandibular Joint Surgery, West China Hospital of Stomatology, Sichuan University, Chengdu, Sichuan, China; 2 West China School of Nursing, Sichuan University, Chengdu, China; 3 School of Stomatology, North Sichuan Medical College, Nanchong, Sichuan, China; 4 State Key Laboratory of Oral Diseases & National Center for Stomatology & National Clinical Research Center for Oral Diseases & Department of Head and Neck Oncology, West China Hospital of Stomatology, Sichuan University, Chengdu, China; 5 Department of Oral and Maxillofacial Surgery, University Clinics of Kinshasa, Faculty of Dental Medicine, University of Kinshasa, Kinshasa, Democratic Republic of Congo

**Keywords:** nomogram, oral cancer, risk prediction model, sleep disturbances, sleep quality

## Abstract

**Objectives:**

This cross-sectional study aimed to develop and validate a predictive model to identify risk factors associated with postoperative sleep disturbances in patients with oral cancer.

**Methods:**

Data were collected from 385 patients with oral cancer who underwent surgery at the Department of Head and Neck Oncology of a tertiary hospital in Sichuan Province between July 2024 and December 2024. Participants were recruited through convenience sampling and allocated to a training group (n = 269) or a validation group (n = 116). The dataset encompassed a comprehensive range of demographic, clinical, and surgical variables, as well as psychometric assessments obtained from validated instruments, including the Pittsburgh Sleep Quality Index (PSQI), MD Anderson Symptom Inventory–Head and Neck Module (MDASI-H&N), Hospital Anxiety and Depression Scale (HADS), and Social Support Rating Scale (SSRS). Model performance was evaluated through reliability testing, intergroup comparisons, and multivariable logistic regression analyses to estimate odds ratios (ORs) and 95% confidence intervals (CIs). A nomogram was subsequently constructed and internally validated.

**Results:**

The incidence of postoperative sleep disturbances was 39.41% in the training set and 39.66% in the validation set. Independent predictors included history of alcohol consumption, longer surgical duration, higher MDASI-H&N and anxiety scores, and lower social support levels. Receiver operating characteristic (ROC) curve analysis demonstrated excellent discrimination, with areas under the curve (AUCs) of 0.902 for the training set and 0.967 for the validation set. Calibration curves showed close agreement between predicted and observed outcomes, with mean absolute errors (MAEs) of 0.0559 and 0.0942, respectively. Decision curve analysis (DCA) further confirmed strong clinical utility within a threshold probability range of 0.0-0.4.

**Conclusion:**

A practical predictive model was developed to identify oral cancer patients at risk of postoperative sleep disturbances. The model achieved strong discrimination, accurate calibration, and notable clinical value.

## 1 Introduction

Oral cancer (OC) is a common malignancy of the head and neck, characterized by aggressive biological behavior, high recurrence rates, and poor prognosis (1). At present, the standard treatment for advanced oral cancer primarily involves surgery in combination with radiotherapy and chemotherapy (2). Owing to the tumor’s anatomical location and the complexity of surgical procedures, patients frequently experience postoperative complications such as pain, dysphagia, and excessive salivation, which can lead to sleep disturbances and markedly impair quality of life (3, 4). Postoperative sleep disturbances (PSD) refer to alterations in sleep architecture and quality during the early postoperative period, typically characterized by reduced or absent rapid eye movement (REM) sleep, increased wakefulness, and sleep fragmentation (5). PSD contributes to delayed wound healing (6), excessive daytime sleepiness, fatigue, prolonged hospitalization, and an increased risk of complications and mortality (7).Sleep disturbances are both prevalent and clinically significant among patients with cancer, with an incidence of 6-20% in the general population (8), rising to approximately 44% among newly diagnosed patients with head and neck cancer (9), and up to 72% in those with advanced disease undergoing treatment, who also exhibit a higher prevalence of obstructive sleep apnea (OSA) (10).Moreover, PSD may cause episodic hypoxemia, hemodynamic instability, and cognitive impairment, further exacerbating postoperative morbidity and mortality (11).

Despite the substantial impact of sleep disturbances, research and management in oral cancer populations remain limited. Most existing studies have focused on more prevalent malignancies such as lung cancer (12)and breast cancer (13), with relatively little attention to postoperative sleep disturbances (PSD) in oral cancer. Hao et al. (14) preliminarily examined the effects of psychological nursing interventions on postoperative sleep quality; however, the small sample size and single-center design limit the generalizability of their findings. Although several international studies have explored potential risk factors for PSD (15–17),the evidence remains inconsistent, and no validated predictive models have yet been established for this population.Perioperative sleep management in cancer care also remains underdeveloped, as many patients underestimate the significance of sleep problems and assume they will resolve spontaneously with recovery (18). This lack of early recognition and intervention further exacerbates sleep disturbances. Notably, PSD in patients with oral cancer tends to be more acute and severe due to the combined effects of surgical airway alterations, xerostomia, and fibrosis resulting from radiotherapy or chemotherapy (4).These challenges, compounded by lifestyle factors such as smoking and alcohol consumption (19, 20),and psychological distress including anxiety and depression (21), substantially increase the risk of both insomnia and hypersomnia. Consequently, these sleep-related issues are closely associated with delayed recovery and a higher incidence of postoperative complications, underscoring the urgent need for targeted, evidence-based interventions.

Therefore, developing a robust predictive model for postoperative sleep disturbances (PSD) in patients with oral cancer is of critical importance. This study employed a cross-sectional design and utilized validated patient-reported outcome measures to determine the incidence and identify key risk factors associated with PSD. Based on these findings, a predictive model was constructed and internally validated to provide empirical evidence supporting the early identification of high-risk individuals and to guide the implementation of targeted, evidence-based interventions in clinical practice.

## 2 Materials and methods

### 2.1 Study setting and participant

This study employed a cross-sectional design with convenience sampling. Participants were patients with oral cancer who underwent surgery at a tertiary hospital in Sichuan Province between July 2024 and December 2024.

Inclusion criteria were as follows: (1) age ≥ 18 years; (2) ability to communicate effectively; (3) histopathological confirmation of oral cancer; (4) receipt of surgical treatment; (5) an anticipated postoperative hospital stay exceeding three days; and (6) voluntary participation with signed informed consent.

Exclusion criteria were: (1) a pre-existing diagnosis of sleep apnea; (2) severe dysfunction of major organs; and (3) auditory, visual, or language impairments that could interfere with questionnaire completion or study participation.

### 2.2 Sample size calculation

The sample size was calculated using the formula for cross-sectional studies (22):


$$N=\frac{Z^{2}\cdot (P\cdot (1-P))}{E^{2}}$$

Considering an anticipated 15% rate of invalid questionnaire returns, the minimum required sample size was calculated to be 256 participants. To enable a 7:3 allocation between the training and validation cohorts, the total required sample size was estimated at 356. Ultimately, 385 participants were enrolled in this study, satisfying the sample size requirements for cross-sectional research.

### 2.3 Measurement

#### 2.3.1 General data survey

The general information questionnaire was developed by the research team following a comprehensive review of the relevant literature. It comprised two sections: (1)sociodemographic variables, including sex, age, body mass index (BMI), marital status, time since diagnosis, occupation, and educational level; (2)disease and treatment-related factors (23), such as preoperative albumin level, surgical duration and complexity, preoperative sleep status, tumor stage and location, history of recurrence, prior radiotherapy or chemotherapy, smoking and alcohol use, comorbidities (e.g., hypertension and diabetes), and surgical procedures, including free tissue flap reconstruction, neck lymphadenectomy, bone resection, tracheostomy, gastric tube placement, and postoperative analgesic use.

For this study, key lifestyle factors were operationally defined as follows. Smoking history was defined as current smoking, indicated by smoking at least one cigarette per day for at least 6 months, or past smoking, defined as a cumulative exposure of at least 5 pack-years with smoking cessation within the past 5 years (19). Alcohol consumption history was defined as current alcohol use, indicated by consumption of at least 50 mL of liquor per day for at least 1 year, or past alcohol use, defined as meeting the same criterion for at least 1 year with cessation within the past 3 years (38).Surgical grade was defined based on surgical complexity and extent of resection, with reference to the American Joint Committee on Cancer (AJCC) Head and Neck Cancer Staging Manual (23) and clinical guidelines for oral and maxillofacial surgery. Procedures were categorized as three-level surgery, defined as local tumor resection without neck lymphadenectomy, including partial glossectomy for T1–T2 tongue cancer and local excision of buccal mucosa cancer, or four-level surgery, defined as extended tumor resection with neck lymphadenectomy, including hemiglossectomy, subtotal or total glossectomy, and tumor resection with free flap reconstruction.

#### 2.3.2 Pittsburgh sleep quality index, PSQI

The Pittsburgh Sleep Quality Index (PSQI), developed by Buysse et al. (24) and translated into Chinese by Liu Xianchen et al. (25), was used to assess sleep quality. The instrument consists of two components: self-report and observer-rated sections. Of these, only the self-report portion contributes to the total score. It evaluates seven dimensions on a 4-point Likert scale, with the total score representing the sum of all dimension scores. The total PSQI score ranges from 0 to 21, with higher scores indicating poorer sleep quality. The validated Chinese version demonstrated good reliability (Cronbach’s α = 0.84).

#### 2.3.3 Anderson symptom inventory-head and neck

The MD Anderson Symptom Inventory-Head and Neck Module (MDASI-H&N) is a self-report instrument developed by the University of Texas MD Anderson Cancer Center to assess symptom severity in patients with cancer (26) and has been widely applied in clinical practice. In this study, the validated Chinese version translated by Wang X.S. et al. (27) was used. The scale comprises two sections, of which only the first was employed. According to the MDASI User Guide (28), each item is rated on a scale from 0 (“no symptom”) to 10 (“worst possible severity”). The mean score of the most severe items represents the overall symptom burden, with higher scores indicating greater symptom severity. The scale demonstrates excellent internal consistency, with a Cronbach’s α coefficient of 0.91.

#### 2.3.4 Hospital anxiety and depression scale

The Hospital Anxiety and Depression Scale (HADS), developed by Snaith and Zigmond in 1983 (29), is widely used to assess psychological distress in clinical populations (30). The scale includes two subscales, anxiety (HADS-A) and depression (HADS-D), with seven items each. Respondents rate the frequency or intensity of symptoms over the past month using a 4-point Likert scale ranging from 0 to 3. Subscale scores are obtained by summing the item scores, with higher values indicating greater anxiety or depression. A cutoff score of ≥8 on either subscale denotes clinically significant symptoms. The HADS demonstrates excellent internal consistency, with a Cronbach’s α coefficient of 0.94.

#### 2.3.5 Social support rating scale

The Social Support Rating Scale (SSRS), developed by Xiao Shui (31), assesses an individual’s perceived level of social support. The scale consists of ten items covering three dimensions: objective support, subjective support, and support utilization. Items 1–5 and 8–10 are rated on a 4-point Likert scale (1-4), while items 6 and 7 are scored according to the number of available support sources, with a score of 0 indicating “no sources of support” and higher scores reflecting a greater number of support sources. The total score is obtained by summing all item scores, with higher totals indicating stronger perceived social support. Based on total score thresholds, social support is categorized as low (≤22 points), moderate (23–44 points), or high (≥45 points). In this study, the SSRS demonstrated good internal consistency, with a Cronbach’s α coefficient of 0.87.

### 2.4 Data collection

**Figure 1:**
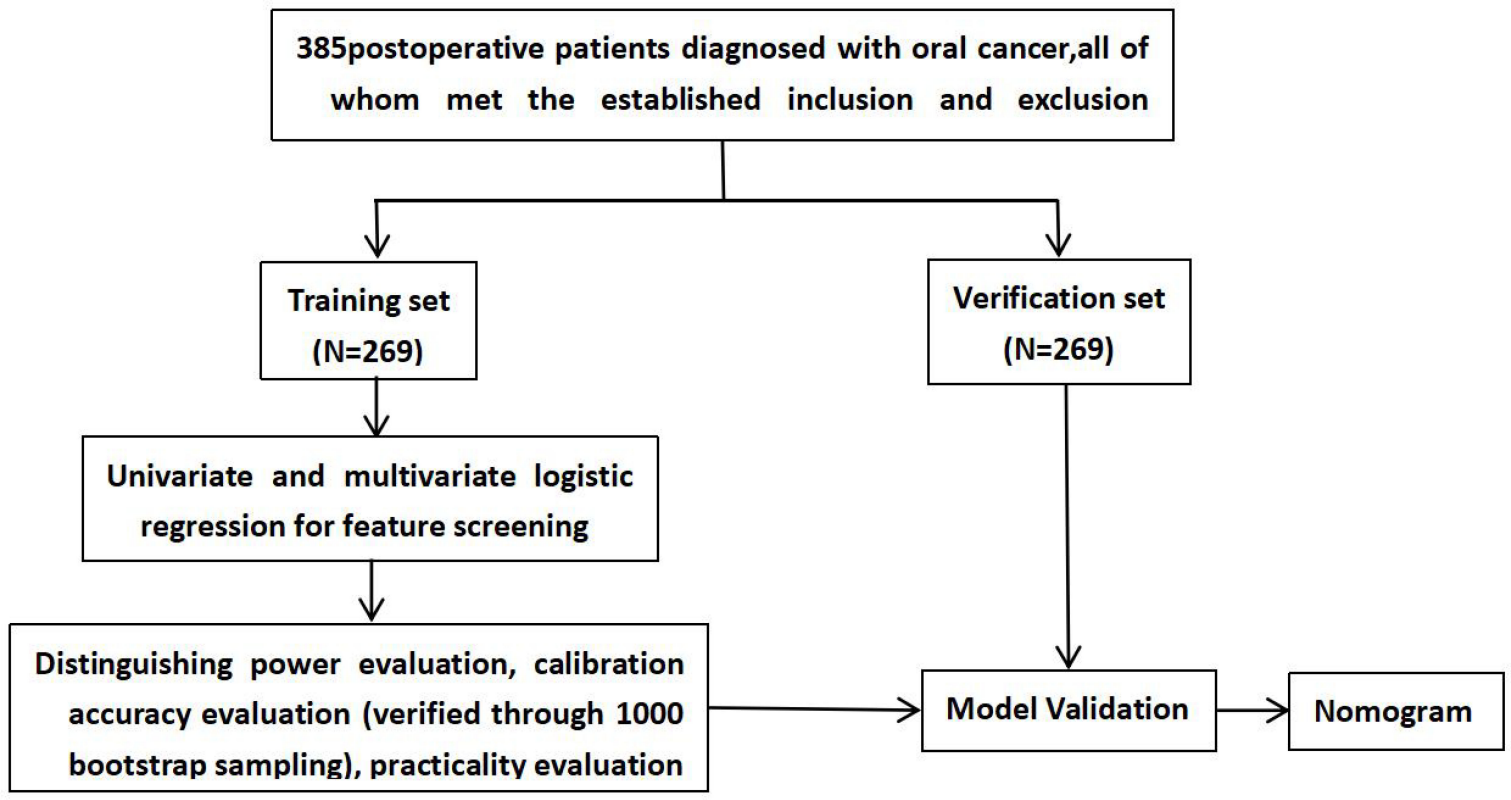
A schematic diagram of model development.

Data were collected using the Wenjuanxing online platform. Upon hospital admission, and after obtaining informed consent, questionnaires were distributed by the research team. The General Data Survey and the Pittsburgh Sleep Quality Index (PSQI) were administered at that time. On postoperative day 3, participants completed the Pittsburgh Sleep Quality Index (PSQI), the MD Anderson Symptom Inventory–Head and Neck Module (MDASI-H&N), the Hospital Anxiety and Depression Scale (HADS), and the Social Support Rating Scale (SSRS). The interval between surgery and assessment was 3 days, with a range of 2–4 days depending on individual postoperative recovery.To ensure data quality and procedural consistency, researchers provided standardized explanations of the study’s purpose, content, and procedures before questionnaire administration. For participants with speech impairments, reading difficulties, or other challenges, the researcher read each item aloud, clarified the content when necessary, and recorded responses according to the participant’s indications while maintaining strict objectivity and neutrality. Additional details are shown in **Figure 1**.

### 2.5 Statistical analysis

Data were analyzed using SPSS version 25.0 (IBM Corp., Armonk, NY, USA) and R version 4.3.3 (R Foundation for Statistical Computing, Vienna, Austria). All statistical tests were two-tailed, and a P value< 0.05 was considered significant. Quantitative variables were assessed for normality and presented as mean ± standard deviation (SD) or median (interquartile range, IQR), as appropriate. Between-group comparisons were performed using independent-samples t tests or Mann-Whitney U tests, while categorical variables were analyzed using χ² tests.

Variables with P< 0.05 in univariate analyses were entered into multivariable logistic regression, with postoperative sleep disturbance as the dependent variable. Odds ratios (ORs) and 95% confidence intervals (CIs) were estimated, and multicollinearity was assessed using the variance inflation factor (VIF< 5).

A predictive nomogram was constructed in R to estimate postoperative sleep disturbance risk. Model performance was evaluated by the area under the receiver operating characteristic (ROC) curve for discrimination, calibration plots for agreement, and decision curve analysis (DCA) for clinical benefit.

### 2.6 Ethical principles

All procedures involving human participants were conducted in accordance with the ethical standards of the institutional and/or national research committees and with the 1964 Declaration of Helsinki and its later amendments, or comparable ethical standards. Ethical approval for this study was obtained from the Ethics Committee of the West China Hospital of Stomatology (Approval No. WCHSIRB-D-2024-271). Written informed consent was obtained from all participants after they were provided with a full explanation of the study objectives and procedures, and all participants voluntarily signed the consent form.

## 3 Results

### 3.1 Basic characteristics of study participants

#### 3.1.1 General data result statistics

**Figure 2:**
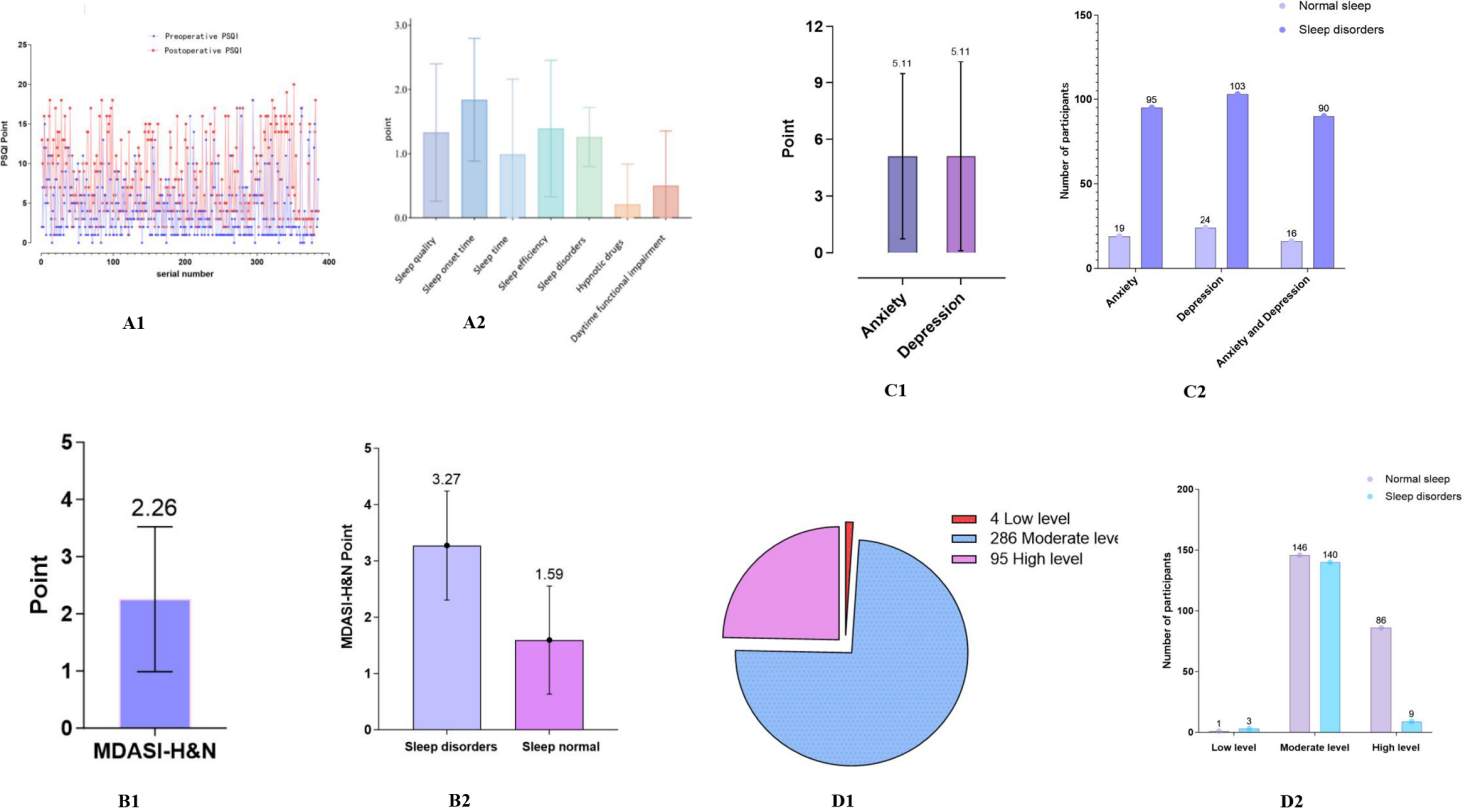
Basic characteristics of study participants. **(A1, A2)** Pittsburgh Sleep Quality Index (PSQI) score; **(B1, B2)** Anderson Symptom Inventory-Head and Neck (MDASI-H&N)score; **(C1, C2)** Hospital Anxiety and Depression Scale result; **(D1, D2)** Social Support Rating Scale (SSRS) results.

A total of 385 patients were included in the study (**Figure 2**), comprising 233 males (60.5%) and 152 females (39.5%). Participants ranged in age from 18 to 93 years, with a mean age of 60.64 ± 13.21 years. Among them, 255 patients (66.2%) reported no history of alcohol consumption. The mean duration of surgery was 3.96 ± 2.19 hours; 199 patients (51.7%) underwent procedures lasting less than 4 hours, whereas 186 patients (48.3%) underwent surgeries lasting 4 hours or longer. Among the 385 patients, 84 (21.8%) received adjuvant treatment, including 47 cases of radiotherapy, 23 cases of chemotherapy, and 14 cases of concurrent chemoradiotherapy. The remaining 301 patients (78.2%) did not undergo adjuvant treatment during the study period. Detailed demographic and clinical characteristics of the study population are summarized in **Table 1**.

**Table 1:** General information of rral cancer patients (n=385).

Variable		Frequency	Percentage(%)
Gender			
	Male	233	60.5
	Female	152	39.5
Age			
	<45years	44	11.4
	45~59years	118	30.6
	≥60years	223	58.0
Smoking History			
	With	153	39.7
	Without	232	60.3
History Of Alcohol Consumption			
	With	130	33.8
	Without	255	66.2
Tumor site			
	Tongue	119	30.9
	Buccal mucosa	96	24.9
	Floor of mouth	20	5.2
	Gingiva	80	20.8
	Palate	22	5.7
	Others	48	12.5
Free tissue flap repair			
	With	250	64.9
	Without	135	35.1
Lymph node dissection of the neck			
	With	271	70.4
	Without	114	29.6
intermaxillary bone			
	With	214	55.6
	Without	171	44.4
Surgical Grade			
	Three-level surgery	51	13.2
	Four-level surgery	334	86.8
Surgical Duration(h)			
	<4h	199	51.7
	≥4h	186	48.3
Adjuvant Treatment			
	With(Radiotherapy/Chemotherapy/Concurrent Chemoradiotherapy)	84	21.8
	Without	301	78.2

#### 3.1.2 Pittsburgh sleep quality index

In this study, the mean preoperative PSQI score among the 385 patients was 4.16 ± 3.75, which increased to 7.51 ± 4.82 after surgery. Overall, 290 patients (75.32%) exhibited a decline in sleep quality following surgery (**Figure A1**), and 152 patients (39.48%) met the criteria for postoperative sleep disturbances. Among the PSQI subdimensions, sleep latency (1.84 ± 0.96), sleep efficiency (1.39 ± 1.06), and overall sleep quality (1.33 ± 1.07) were the most adversely affected (**Figure A2**).

#### 3.1.3 Anderson symptom inventory-head and neck

The mean MDASI-H&N score among all 385 patients was 2.26 ± 1.27 (**Figure B1**). Patients with postoperative sleep disturbances had a significantly higher mean score (3.27 ± 0.97) compared with those without sleep disturbances (1.59 ± 0.96) (**Figure B2**).

#### 3.1.4 Hospital anxiety and depression scale

Among the 385 patients, the mean scores on the Hospital Anxiety and Depression Scale (HADS) were 5.11 ± 4.37 for anxiety (HADS-A) and 5.11 ± 5.01 for depression (HADS-D) (**Figure C1**). Postoperatively, 114 patients (29.61%) exhibited symptoms of anxiety, while 127 patients (32.99%) showed symptoms of depression (**Figure C2**).

#### 3.1.5 Social support rating scale

According to the Social Support Rating Scale (SSRS) results, among the 385 patients, 4 (1.03%) reported low levels of social support, 286 (74.29%) had moderate levels, and 95 (24.68%) had high levels (**Figure D1**). Postoperative sleep disturbances (PSD) occurred in 75.00% of patients with low social support, 48.95% of those with moderate support, and only 9.47% of those with high support (**Figure D2**).

### 3.2 Data analysis

#### 3.2.1 Baseline Comparison

The 385 patients were divided into a training set (July 2024 to October 2024) and a validation set (November to December 2024) in a 7:3 ratio based on admission dates. No statistically significant differences were observed in baseline characteristics between the two groups (**Table 2**).

**Table 2:** Baseline comparison between the model training and validation sets (n = 385).

Variable		Training set (n=269)	Validation set (n=116)	*t*/*Z/x^2^*	*P*
Gender	Female	102 (37.92)	50 (43.10)	0.912	0.340
	Male	167 (62.08)	66 (56.90)		
Surgical Grade	Three-levelsurgery	41 (15.24)	10 (8.62)	3.092	0.079
	Four-level surgery	228 (84.76)	106 (91.38)		
Surgical Duration (h)		3.90 (2.33,5.53)	3.91 (2.32,5.22)	-0.745	0.456
Smoking History	No	159 (59.11)	73 (62.93)	0.495	0.482
	Yes	110 (40.89)	43 (37.07)		
History Of Alcohol Consumption	No	175 (65.06)	80 (68.97)	0.554	0.457
	Yes	94 (34.94)	36 (31.03)		
Anxiety	No	192 (71.38)	79 (68.10)	0.416	0.519
	Yes	77 (28.62)	37 (31.90)		
Depression	No	181 (67.29)	77 (66.38)	0.030	0.862
	Yes	88 (32.71)	39 (33.62)		
MDASI-H&N		48.00 (28.00,71.00)	43.50 (18.25,73.75)	-1.568	0.117
Social Support Level	Low Level	3 (1.12)	1 (0.86)	1.297	0.523
	Moderate level	204 (75.83)	82 (70.69)		
	High Level	62 (23.05)	33 (28.45)		

MDASI-H&N:express Anderson Symptom Inventory-Head and Neck score.

#### 3.2.2 Univariate analysis of the model training set

In the training set, patients were categorized into two groups based on the presence or absence of postoperative sleep disturbances: the sleep disturbance group (n = 106) and the normal sleep group (n = 163). Univariate analysis revealed significant associations between postoperative sleep disturbances and gender (P = 0.018), MDASI-H&N score (P< 0.001), surgical duration (P< 0.001), type IV surgery (P = 0.013), history of alcohol consumption (P = 0.019), anxiety (P< 0.001), depression (P< 0.001), and social support level (P< 0.001). Detailed results are presented in **Table 3**.

**Table 3:** Variables with statistically significant differences in the training set (n = 269).

Variable		Sleep disorder group (n=106)	The normal sleep group (n=163)	*t*/*Z/x^2^*	*P*
Gender	Female	31(29.25)	71(43.56)	5.590	0.018^*^
	Male	75(70.75)	92(56.44)		
MDASI-H&N		3.18(2.76,3.74)	1.74 (0.95,2.27)	12.140	<0.001^**^
Surgical Duration(h)		5.13(3.50,6.75)	3.12(1.95,4.68)	-5.966	<0.001^**^
Surgical Grade	Three-level surgery	9(8.49)	32(19.63)	6.172	0.013*
	Four-level surgery	97(91.51)	131(80.37)		
Smoking History	No	54(50.94)	105(64.42)	4.824	0.028*
	Yes	52(49.06)	58(35.58)		
History Of Alcohol Consumption	No	60(56.60)	115(70.55)	5.497	0.019*
	Yes	46(43.40)	48(29.45)		
Anxiety	No	43(40.57)	149(91.41)	81.274	<0.001^**^
	Yes	63(59.43)	14(8.59)		
Depression	No	37(34.91)	144(88.34)	83.327	<0.001^**^
	Yes	69(65.09)	19(11.66)		
Social Support Level	Low Level	2(1.89)	1(0.61)	24.176	<0.001^**^
	Moderate Level	96(90.56)	108(66.26)		
	High Level	8(7.55)	54(33.13)		

*P<0.05; **P<0.001; MDASI-H&N, express Anderson Symptom Inventory-Head and Neck score.

#### 3.2.3 Multivariate regression analysis of the model training set

Postoperative sleep disturbance in oral cancer patients was defined as the dependent variable. Nine variables that showed significant associations in univariate analysis (P< 0.05), including gender and the total MDASI-H&N symptom score, were entered as independent variables and appropriately coded (**Table 4**). Multivariate stepwise logistic regression analysis was then performed to identify independent predictors of postoperative sleep disturbances.

**Table 4:** Assignment of Independent Variables.

Variable Code	Independent variable	Assignment
X_1_	Gender	Female=0;Male=1
X_2_	Surgical Grade	Three-level surgery=0;Four-level surgery=1
X3	Smoking History	No=0;Yes=1
X4	History Of Alcohol Consumption	No=0;Yes=1
X5	Anxiety	No=0;Yes=1
X6	Depression	No=0;Yes=1
X7	Social Support Level	Low Level=1 Moderate Level=2 High Level=3
X_8_	Surgical Duration(h)	Original Value
X_9_	MDASI -H&N	Original Value

MDASI -H&N, express Anderson Symptom Inventory-Head and Neck score.

Five variables remained in the final model: history of alcohol consumption, surgical duration, total MDASI-H&N score, anxiety, and social support level. Collinearity diagnostics indicated that all variables had variance inflation factors (VIFs) below 5, confirming the absence of significant multicollinearity. Among these, history of alcohol consumption (P = 0.009, OR = 2.670), surgical duration (P = 0.002, OR = 1.292), total MDASI-H&N score (P< 0.001, OR = 2.768), and anxiety (P< 0.001, OR = 5.780) were identified as independent risk factors for postoperative sleep disturbances, whereas social support level (P = 0.017, OR = 0.319) served as a protective factor (**Table 5**).

**Table 5:** Multivariate Logistic Regression Analysis of the Training Set.

Variable	B	SE	Wald	*P*	OR (95% CI)	VIF
History Of Alcohol Consumption	0.982	0.373	6.923	0.009	2.670(1.285~5.548)	1.021
Surgical Duration(h)	0.256	0.083	9.577	0.002	1.292(1.099~1.520)	1.171
MDASI -H&N	1.018	0.196	26.857	<0.001	2.768(1.883~4.068)	1.633
Anxiety	1.754	0.418	17.582	<0.001	5.780(2.546~13.123)	1.484
Social Support Level	-1.141	0.479	5.673	0.017	0.319(0.125~0.817)	1.116
Intercept	-2.404	1.164	4.265	0.039	0.090	–

MDASI -H&N, express Anderson Symptom Inventory-Head and Neck score; B, Regression Coefficient; SE, Standard Error; Wald, statistical magnitude; P, P - value; OR, Odds ratio; 95% CI, 95% Confidence interval; VIF, Variance Inflation Factor.

#### 3.2.4 Subgroup analysis of adjuvant treatment

To examine the association between adjuvant treatment and postoperative sleep disturbances, a subgroup analysis was conducted. The incidence of postoperative sleep disturbances was 45.2% (38/84) in patients with adjuvant treatment and 38.2% (114/301) in those without. Univariate analysis showed no significant association (χ² = 1.864, P = 0.172). Multivariate logistic regression (adjusting for alcohol consumption history, surgical duration, MDASI-H&N score, anxiety, and social support level) confirmed that adjuvant treatment was not an independent predictor (OR = 1.215, 95% CI: 0.703–2.098, P = 0.483).

### 3.3 Development of the risk prediction model

Based on the results of the multivariate logistic regression analysis, a predictive model for postoperative sleep disturbances in oral cancer patients was established. The probability of developing postoperative sleep disturbances (PSD) was calculated using the following equation:

Logit(p) = -2.404 + 0.982 * X4 + 1.754 * X5 - 1.141 * X7 + 0.256 * X8 + 1.018 * X9

where:X4 is history of alcohol consumption,X5 is Anxiety,X7 is Social Support Level,X8 is Surgical Duration(h),X9 is the MDASI-H&N.

### 3.4 Evaluation and validation of the risk prediction model

#### 3.4.1 Discrimination evaluation

**Figure 3:**
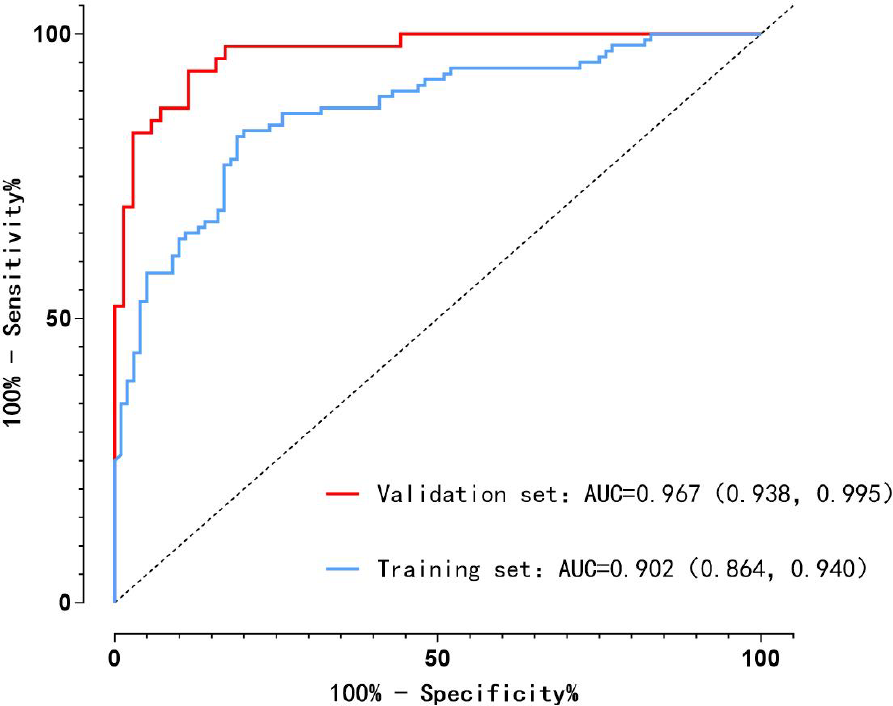
ROC curve of the prediction model, ROC curves for training/validation sets showed excellent discriminative ability, AUC = 0.902 (95% CI: 0.864–0.940) in training set, 0.967 (95% CI: 0.938–0.995) in validation set (both > 0.90), confirming outstanding performance in identifying high-risk patients.

The receiver operating characteristic (ROC) curves for both the training and validation sets (**Figure 3**) demonstrated excellent discriminative ability. The area under the curve (AUC) was 0.902 (95% CI: 0.864–0.940) in the training set and 0.967 (95% CI: 0.938–0.995) in the validation set, both exceeding 0.90. These findings indicate that the predictive model achieved outstanding performance in distinguishing patients at risk of postoperative sleep disturbances.

#### 3.4.2 Calibration evaluation

The Hosmer–Lemeshow goodness-of-fit test yielded a χ² value of 10.269 (df = 8, P = 0.247), indicating satisfactory model calibration. The predicted probabilities closely aligned with the observed incidence of postoperative sleep disturbances, showing no significant deviation between predicted and actual outcomes. These results confirm that the model demonstrates strong reliability and accurate calibration in estimating postoperative sleep disturbance risk among patients with oral cancer.

**Figure 4:**
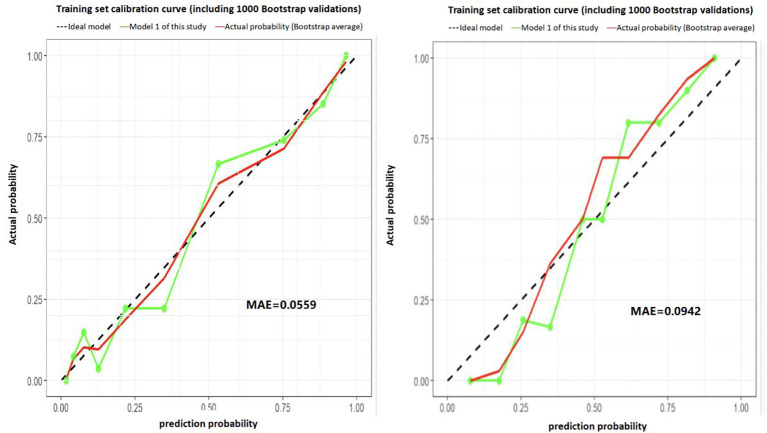
Calibration curve of the prediction model, Internal validation calibration curve (1,000 Bootstrap Resamples), calibration curve showed strong agreement between predicted/observed probabilities: training set MAE = 0.0559, validation set MAE = 0.0942. Green calibration line closely overlapped red reference and black ideal lines, confirming robust calibration performance and high internal reliability for oral cancer patients’ postoperative sleep disturbance risk estimation.

The calibration curve, generated using 1,000 bootstrap resamples for internal validation (**Figure 4**), demonstrated strong agreement between predicted and observed probabilities. The mean absolute error (MAE) was 0.0559 for the training set and 0.0942 for the validation set. The calibration line (green) closely overlapped both the reference (red) and ideal (black) lines, indicating excellent consistency between predicted and actual outcomes. These findings confirm that the model exhibits robust calibration performance and high internal reliability.

#### 3.4.3 Clinical utility evaluation

**Figure 5:**
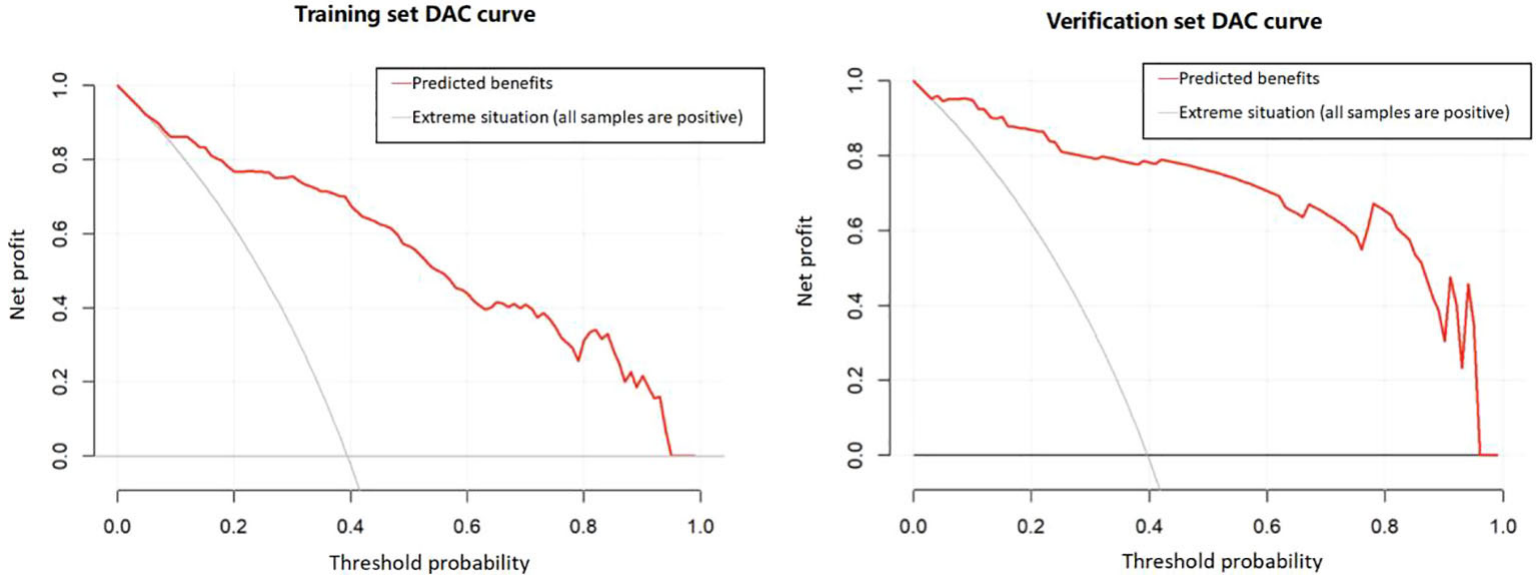
Decision Curve of the Prediction Model,Model yielded substantial net clinical benefit at threshold probability 0–0.4. It aids in identifying high-risk patients for targeted interventions (reducing overtreatment/missed diagnoses), enhancing postoperative sleep management in oral cancer patients.

The decision curve analysis (DCA) for both the training and validation sets (**Figure 5**) demonstrated that the model provided a substantial net clinical benefit across a threshold probability range of 0–0.4, indicating strong clinical applicability. This result suggests that the model effectively assists clinicians in identifying high-risk patients who may benefit from targeted interventions, thereby reducing both overtreatment and missed diagnoses. Collectively, these findings highlight the model’s robust predictive performance and its potential to enhance postoperative sleep management in patients with oral cancer.

### 3.5 Visualization of the risk prediction model

**Figure 6:**
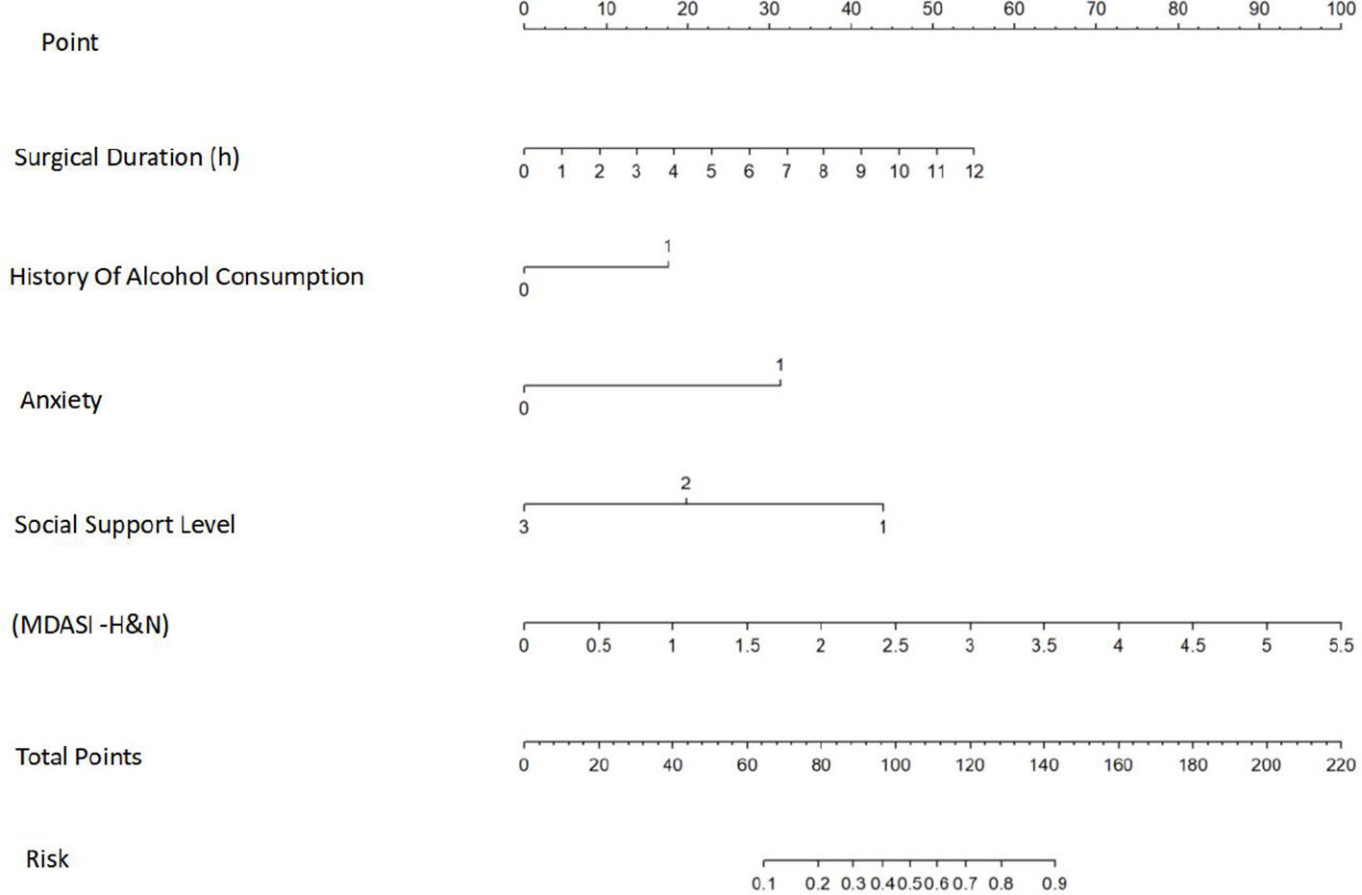
Nomogram of the Prediction Model,Built in R 4.3.3 using multivariate logistic regression-derived independent predictors. Enables clinicians to accurately estimate individual PSD risk for early high-risk identification and personalized postoperative care.

Independent predictors identified through multivariate logistic regression were incorporated into R version 4.3.3 to construct a visual nomogram for predicting postoperative sleep disturbances (PSD) in patients with oral cancer (**Figure 6**). The resulting nomogram provides an intuitive, evidence-based tool that enables clinicians to accurately estimate individual PSD risk, facilitating early identification of high-risk patients and supporting personalized postoperative management.

For instance, an oral cancer patient with a history of alcohol consumption, a surgical duration of 2 hours, a mean MDASI-H&N score of 2.27, the presence of anxiety, and a moderate level of social support would receive the following nomogram scores: history of alcohol consumption (18 points), surgical duration (10 points), MDASI-H&N score (42 points), anxiety (30 points), and social support (20 points), resulting in a total of 120 points. This total corresponds to an estimated 70% probability of developing postoperative sleep disturbances.

## 4 Discussion

This study developed the first visual predictive model for postoperative sleep disturbances (PSD) in patients with oral cancer. Among 385 participants, 75.3% experienced a decline in sleep quality and 39.5% met the criteria for PSD, consistent with prior research. Difficulties in sleep initiation and reduced sleep efficiency were the most frequently reported symptoms. History of alcohol consumption, longer surgical duration, higher symptom burden, and elevated anxiety were identified as independent risk factors, whereas stronger social support served as a protective factor. The model demonstrated excellent discriminative ability (AUC > 0.90), good calibration, and strong clinical applicability. The resulting nomogram provides a rapid and user-friendly tool for individualized postoperative risk assessment and targeted intervention planning.

From a mechanistic perspective, multiple physiological and psychosocial factors jointly contribute to postoperative sleep disturbances in patients with oral cancer.

Both symptom severity (MDASI-H&N) and anxiety were identified as significant predictors of postoperative sleep disturbances. A greater symptom burden and higher anxiety levels were associated with an increased risk of sleep disturbances, consistent with the findings of Grayson et al. (32). Moreover, a bidirectional vicious cycle exists between the two, consistent with findings from previous studies (33).Symptoms following radiotherapy, such as speech and swallowing difficulties, xerostomia, gastric tube placement, and reflux, may exacerbate malnutrition and irritability (34).Nutritional deficits may further impair neurotransmitter synthesis, leading to fragmented sleep patterns (35).Excessive anxiety activates neuroendocrine stress responses that disrupt circadian rhythms and heighten physiological arousal, effects that may be compounded by concerns regarding surgical outcomes and limited social support (36).In practice, comprehensive rehabilitation targeting oral and swallowing function, coupled with patient–family education and psychological counseling, is recommended to alleviate discomfort, stabilize emotional responses, and enhance postoperative sleep quality.

History of alcohol consumption was identified as a significant risk factor for postoperative sleep disturbances, consistent with previous studies (37, 38). Chronic alcohol consumption disrupts neurotransmitter homeostasis, reduces upper airway muscle tone, and induces withdrawal-related arousal, collectively leading to altered sleep architecture and fragmentation (39).This cyclical pattern of consumption, withdrawal, and insomnia may further exacerbate postoperative sleep disruption. From a clinical perspective, early implementation of motivational interviewing during hospitalization, combined with counseling on moderation, balanced nutrition, and maintenance of regular sleep-wake routines, may help mitigate alcohol-related sleep impairments and promote postoperative recovery (40).

Longer surgical duration was positively associated with postoperative sleep disturbances. Although few studies have directly examined this relationship, Pettit et al. (41) reported that extended operative times are correlated with poorer postoperative outcomes, indirectly supporting our findings. Prolonged anesthesia may disrupt circadian rhythms and melatonin metabolism, while heightened physiological stress and inflammatory responses exacerbate postoperative discomfort, creating a self-reinforcing cycle of stress, sleep disturbance, and hormonal imbalance (42, 43). As demonstrated in our previous study (44), aromatherapy effectively enhances parasympathetic activity and stimulates melatonin secretion, representing a feasible, non-pharmacological strategy to improve postoperative sleep quality.Similarly, white noise exposure (45) may promote parasympathetic activation, regulate melatonin release, and further support sleep restoration.

Social support emerged as a protective factor against postoperative sleep disturbances, consistent with previous research (46). Multidimensional support—including informational, instrumental, emotional, and evaluative components—facilitates physical activity, alleviates postoperative symptoms, and alleviates psychological and financial stress. Among these, emotional support demonstrates the strongest association with improved sleep quality (47, 48). Experimental evidence further indicates that social isolation can exacerbate tumor-related inflammatory responses, indirectly compromising overall well-being (49). Clinically, fostering patient engagement in social interactions and physical activities, as well as implementing a patient–caregiver dyadic approach, may strengthen emotional bonds and improve postoperative sleep outcomes.

Building on the proposed mechanisms and predictive model, this study presents a visual nomogram that enables multidimensional, collaborative strategies tailored to individual risk factors. Its strong clinical utility addresses the common challenges of complexity and poor implementation in traditional assessments. By providing a practical, evidence-based framework, the nomogram supports precision nursing and facilitates targeted, patient-centered interventions.

This study has several limitations. First, the sample was obtained from a single tertiary hospital in Sichuan Province, which may limit the generalizability of the findings due to the absence of a multicenter design. Second, the cross-sectional design precludes assessment of the long-term trajectory of postoperative sleep disturbances. Third, the lack of endocrine and other biological markers may have led to incomplete identification of underlying risk factors. Future studies should adopt multicenter cohort designs, integrate objective biological indicators, and refine the model to enable seamless integration of risk prediction with timely clinical interventions.

Adjuvant treatment was not identified as an independent risk factor for postoperative sleep disturbances in the present study. Although patients receiving adjuvant treatment showed a slightly higher incidence of sleep disturbances, the difference was not statistically significant. This may be related to the limited sample size of patients undergoing adjuvant treatment and the influence of other factors already included in the multivariate model. Moreover, sleep disturbances were assessed on the third postoperative day, before most patients initiated adjuvant treatment, which may have limited the detection of treatment-related effects.

## 5 Conclusion

This study found a 39.48% incidence of postoperative sleep disturbances among patients with oral cancer, highlighting the urgent need to improve sleep management in this population. Alcohol use history, longer surgical duration, higher MDASI-H&N scores, and elevated anxiety were identified as independent risk factors, whereas greater social support served as a protective factor. The resulting nomogram demonstrated excellent discrimination (AUC > 0.9), strong calibration, and high clinical utility. This predictive tool enables early identification of high-risk patients and supports the implementation of personalized, evidence-based sleep interventions in clinical practice.

## Data Availability

The datasets presented in this study can be found in online repositories. The names of the repository/repositories and accession number(s) can be found in the article/supplementary material.
